# Gamification and Control of Nitinol Based Ankle Rehabilitation Robot

**DOI:** 10.3390/biomimetics6030053

**Published:** 2021-09-21

**Authors:** Chong Tune Hau, Darwin Gouwanda, Alpha A. Gopalai, Cheng Yee Low, Fazah A. Hanapiah

**Affiliations:** 1 School of Engineering, Monash University Malaysia, Bandar Sunway 47500, Selangor, Malaysia; chong.tune.hau@monash.edu (C.T.H.); alpha.agape@monash.edu (A.A.G.); 2 Faculty of Mechanical and Manufacturing Engineering, Universiti Tun Hussein Onn Malaysia, Parit Raja, Batu Pahat 86400, Johor, Malaysia; cylow@uthm.edu.my; 3 Faculty of Medicine, Universiti Teknologi MARA, Sungai Buloh 47000, Selangor, Malaysia; fazahakhtar@yahoo.com

**Keywords:** ankle rehabilitation, rehabilitation robot, nitinol, rehabilitation game

## Abstract

Conventional ankle rehabilitation exercises can be monotonous and repetitive. The use of robots and games can complement the existing practices, provide an engaging environment for the patient and alleviate the physiotherapist’s workload. This paper presents an ankle rehabilitation robot that uses two nitinol wire actuators and a Pong game to provide foot plantarflexion and dorsiflexion exercises. Nitinol is a type of smart material that has high volumetric mechanical energy density and can produce translational motion. A two-state discrete antagonistic control is proposed to manipulate the actuators. The system was tested on healthy participants and stroke patients. The results showed that the robot was safe and compliant. The robot did not forcefully plantarflex or dorsiflex the foot when the participant exerted opposing force. The actuators worked antagonistically to flex to the foot as intended, in sync with the up and down motions of the player’s bat in the game. These behaviors demonstrated the feasibility of a nitinol-based ankle rehabilitation robot and a simple and yet intuitive game in providing interactive rehabilitation exercise. The robot is expected to enhance the patient’s experience, participation and compliance to the rehabilitation routine and to quantitatively monitor the patient’s recovery progress.

## 1. Introduction

Ankle rehabilitation is a laborious and costly treatment, and it involves commitments from both patients and physiotherapists. The patient has to actively participate and comply with the prescribed treatment while the physiotherapist has to manually assess and perform the repetitive rehabilitation routine. However, the assessment can be subjective based on the therapist’s knowledge and experience. The ankle rehabilitation robot can address these limitations and offer several advantages. The robot can provide different types of exercise in one system, including passive and active rehabilitation exercises. It can also provide a rich stream of information to assist in diagnosis, prognosis and assurance of patients’ compliance with the treatment and maintenance of patients’ records. Lastly, it also reduces the load on physiotherapists so that they can now focus on performance monitoring and planning without having to worry about the menial portions of the rehabilitation.

Ankle rehabilitation robots can be categorized into two major groups: Platform robots and wearable robots [1]. The platform robots are stationary robots that have a moveable platform. These robots tend to be bulky and require space for storage and operation. Some of the popular platform robots are Rutgers Ankle [2], CARR (Compliant Ankle Rehabilitation Robot) [3,4], the parallel manipulator robots [5,6,7] and other robots proposed by [8,9,10]. By being stationary, these robots cannot be used for gait training. On the contrary, the wearable robots are light, compact and are mainly developed to help the patients in gait training by correcting their foot orientation. The popular wearable robots are the ankle–foot orthosis (AFO) proposed by [11,12,13,14].

Parallel manipulator robots are commonly adopted to provide stationary rehabilitation exercises for the knee [15,16,17] and ankle [2,3,4,5,6,7,8,9,10,11,12,13,14]. It has a simple and compact construction and can generate movement of up to 6 degrees of freedom [18]. The dynamic behavior of this robot is well established and can be derived from the classical closed-loop kinematic chain approach. It also has a rigid structure and accurate movement; therefore, it is safe for rehabilitation purposes. Zhang et al. [4] developed CARR with reconfigurable workspace and torque capacity to provide two different sets of motion: ankle plantarflexion–dorsiflexion, inversion–eversion and internal–external rotation; ankle plantarflexion–dorsiflexion and inversion–eversion. Valles et al. [6] designed a parallel robot that uses three linear actuators to perform active, active-assistive and active-resistive exercises. Jamwal et al. [7] developed an ankle rehabilitation robot that uses two pairs of pneumatic artificial muscles. The robot can adjust its impedance based on the user’s participation such that the robot assistance decreases as the user’s participation increases.

The use of different materials and actuations has been an emerging trend in robotics. Nitinol (a combination of nickel and titanium), also known as shape memory alloy, is one of the materials that is being explored and used extensively in soft robots [19,20,21,22,23] and rehabilitation robots [24]. It has high power to weight ratio and the ability to remember its shape after deformation. When nitinol temperature increases, it transforms from martensite state to austenite state, returns to its original shape, and generates a pulling force that is several times larger than its own weight. Vice versa, when the temperature decreases, it transforms from austenite to martensite state and becomes easily deformed by an external force. Nitinol is known for its low recoverable strain and cooling rate. In the previous work [25], the nitinol wire actuator was designed and developed to overcome the first limitation. The actuator has multiple discs that amplify the Nitinol wire strain and can generate a linear displacement of 26 mm. A periodical cooling was proposed and integrated into the robot to induce forced convection to the actuator. This approach addresses the second limitation. However, this robot has a rudimentary control mechanism, and it can only provide passive rehabilitation exercise that involves continuous foot dorsiflexion and plantarflexion.

The use of the game as one of the rehabilitation tools has gained traction in recent years. Gamification of the rehabilitation exercise can complement the existing practices that commonly involve monotonic and repetitive dorsiflexion and plantarflexion at the preset duration, frequency and range of motion (RoM). Related studies showed that gamification of the rehabilitation routines could increase patient’s participation and enhance recovery [26,27,28,29].

Therefore, this paper proposes the gamification and control of an ankle rehabilitation robot that uses nitinol wire actuators. Pong, a classic computer game [30], is implemented in this work. It is a simple and intuitive game. When the foot moves, the player’s bat moves. A two-state discrete antagonistic control was proposed and integrated into the game. It manipulates the movement of the actuators so that they work in tandem to tilt the foot plate of the robot up and down, which translates to the foot dorsiflexion and plantarflexion. The combination of these two methods is expected to enhance patient’s experience, engagement and compliance to the prescribed rehabilitation program and consequently improve the recovery progress.

This paper is arranged as follows: Chapter 2 describes the design of the ankle rehabilitation robot. It also discusses the gamification and control of the robot and the details of the experiment on healthy subjects and stroke patients. Chapter 3 discusses the experiment results and the overall performance of the. Chapter 4 describes the merits and limitations of the robot and some of the possible methods to overcome them in the future.

## 2. Ankle Rehabilitation Robot

**Figure 1:**
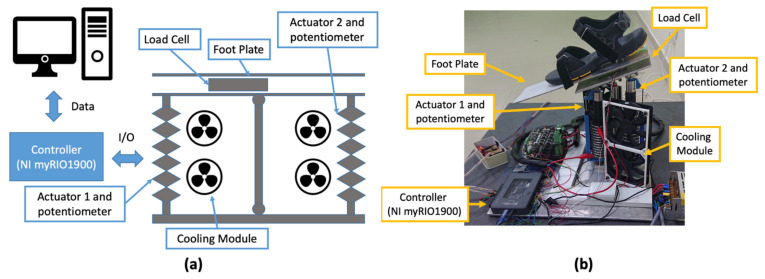
(**a**) Basic schematic of the ankle rehabilitation robot, (**b**) the final working prototype.

This work uses a platform-type ankle rehabilitation robot developed in [25]. It is a stationary robot that has a dimension of 400 × 400 × 298.1 mm (Figure 1a). The RoM of the robot is 25° plantarflexion and 25° dorsiflexion with a maximum payload capability of 27 N. The motion of the robot is controlled by two nitinol wire actuators. The actuator located at the forefoot is denoted as Actuator 1, and the actuator located at the heel is denoted as Actuator 2. Both actuators work antagonistically to plantarflex and dorsiflex the foot. When Actuator 1 contracts and Actuator 2 extends, it tilts the foot plate downward and plantarflexes the foot. Vice versa, when Actuator 2 contracts and Actuator 1 extends, it tilts the foot plate upward and dorsiflexes the foot. The actuator is connected to a DC power driver (MDS40B, Cytron Technologies, Bukit Mertajam, Malaysia) that uses a PWM signal from the controller to manipulate the actuator movement.

The robot uses an integrated cooling module to induce forced convection to the actuator. This module has two fans that are arranged vertically next to the actuator (Figure 1a). Each fan (ShenZhen WeiYe Electronics Co., Ltd., China) has the dimension of 120 × 120 mm and produces 1.876 m^3^/min at 2000 rpm. Two relay switches are used to activate the cooling module. The controller controls the switching of the relays, which activates and deactivates the cooling module.

The robot uses myRIO I900 (National Instrument, Inc., Austin, TX, USA) as the controller and the measurements from a straight bar strain gage load cell and linear potentiometers as the main inputs. The load cell is positioned below the foot plate. It was set up and calibrated such that the foot plate behaves like a cantilever beam. When a downward force is applied at the front of the plate, and the load cell bends downward, it generates positive voltage. Vice versa, when the load cell bends upward, it generates negative voltage. By utilizing this property, the robot can detect the intended motion of the foot: bending downward corresponds to foot plantarflexion, bending upward corresponds to foot dorsiflexion. The linear potentiometer (Honeywell International, Inc., Charlotte, NC, USA) is used to measure actuator displacement during contraction and extension. It has a measuring range of 76.2 mm with a step size of 0.1 mm and a total resistance of 4500 Ω. The potentiometer is also used to determine the orientation of the foot plate.

### 2.1. Nitinol Wire Actuator

**Figure 2:**
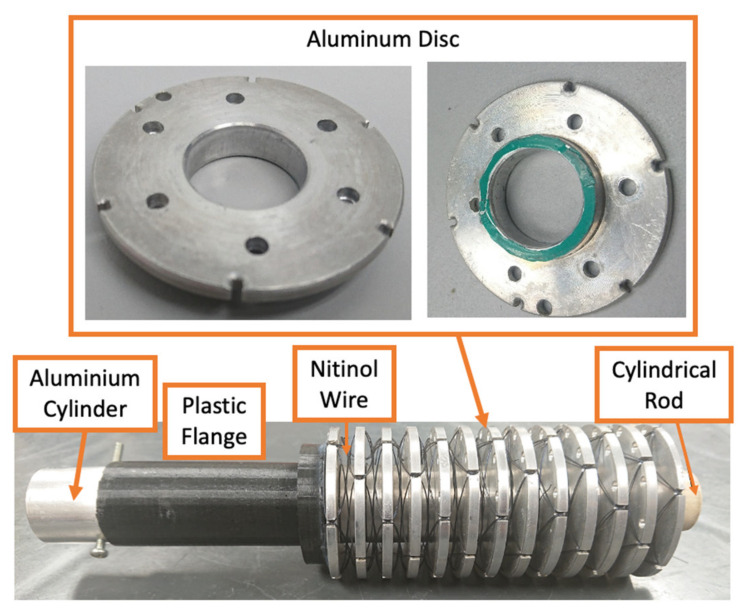
Nitinol wire actuator.

The nitinol wire actuator utilizes the transformation of the wire from the martensite state (low temperature) to austenite state (high temperature) to generate linear force and displacement. The actuator has multiple 50 mm diameter discs and a cylindrical rod at the center, acting as the guide for the discs (Figure 2). The aluminum disc has six notches that are equally spaced around the edge to hold the wire. The nitinol wire is weaved around the disc through these notches in a counter-rotating helical pattern from the first disc to the last disc, repetitively, until all six notches are covered. The actuator is similar to the design by [31], with several unique distinctions. The actuator built for the ankle rehabilitation robot is larger with a diameter of 50 mm and a length of 183.5 mm. It uses a longer 250 µm nitinol wire. It can generate a linear displacement of 38 mm and a force of 27 N. The discs are made of aluminum so that they can dissipate the heat quickly when the nitinol wire transforms from an austenite state to a martensite state.

At the martensite state, the wire is malleable. A bias force is required to extend the actuator. Several studies proposed the use of spring [31,32,33]. In this work, the bias force is produced by the opposing actuator. When one of the actuators contracts, it generates linear force to extend the other actuator.

### 2.2. Two States Discrete Antagonistic Actuator Control

**Figure 3:**
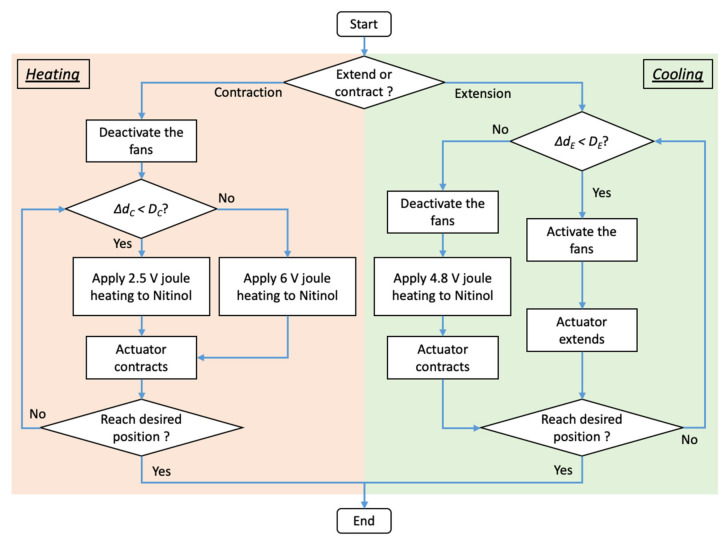
The flowchart of the two-state discrete antagonistic actuator control.

Two states of discrete antagonistic actuator control are proposed to manipulate the position of the nitinol wire actuators: Heating state and cooling state (Figure 3). This method generates antagonistic motion. If one of the actuators operates in the heating state and undergoes contraction, the other actuator operates in a cooling state and undergoes extension.

During the heating state, joule heating is applied to the nitinol wire and transforms the wire from martensite state to austenite state. This transformation shortens the wire and contracts the actuator. This state has two voltage levels: high voltage level (6 V) and low voltage level (2.5 V). Based on the difference between the desired and actual positions (Δ*d_C_*), the controller determines if the actuator operates at a high or low voltage level. A high voltage level is selected if the difference is larger than a preset threshold (*D_C_*) of 0.5 mm. High electrical power is supplied to the nitinol wire to rapidly generate large actuator displacement. A low voltage level is selected if the difference is less than *D_C_*. Low electrical power is supplied to generate small actuator displacement at a slower speed. During contraction, the fans are deactivated to facilitate a faster heating rate.

In the cooling state, the controller activates the fans to induce force convection to the actuator. This rapidly decreases the wire temperature and transforms it from an austenite state to a martensite state. At the martensite state, the wire is malleable, thus allows the actuator to be pulled by the antagonistic actuator. If the difference between the desired and actual positions (Δ*d_E_*) is greater than the threshold (*D_E_*) of 0.1 mm, 4.8 V Joule heating will be applied to the nitinol wire. This restores the wire to an austenite state, contracts the actuator and pulls the other actuator to maintain the position of the foot plate at the desired level.

### 2.3. PONG Game

Pong game is a classic computer game in which the player plays against the opponent (computer) by moving the bat to prevent the ball from entering the scoring region. It is selected because the up and down motions of the bat resemble the foot plantarflexion and dorsiflexion. This work uses the Pong game reported in [30]. Instead of EMG signals from the gastrocnemius and tibialis anterior muscles, the robot uses the position of the actuator and the force applied to the foot plate to control the bat.

**Figure 4:**
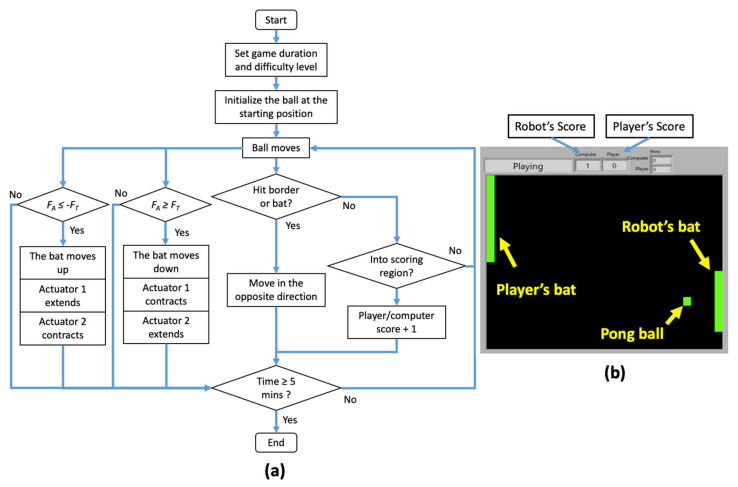
(**a**) The flowchart and (**b**) screenshot of the Pong game.

Figure 4 displays the Pong game in action. The green color ball is initiated at the center of the rectangular box. A set of randomized numbers is generated to determine the ball’s initial speed and direction. When the ball moves and hits the border or the bat, it bounces and travels in the opposite direction. The computer reaction and the speed of the ball can be adjusted to change the difficulty of the game. As the user is getting familiar and better over time, the difficulty can be increased proportionally to challenge the player.

The player’s bat has two positions—upper half and lower half positions, which correspond to the foot dorsiflexion and plantarflexion, respectively. At the start of the game, the bat is positioned at the center. This location is in relation to the robot’s neutral position; as the foot plate tilts upward, the bat moves to the upper half position and vice versa. Doing so reduces the complexity of the game, thus allowing a patient with a severe condition to play the game without much difficulty.

The Pong game uses measurements from the load cell to move the bat up and down (Figure 4). If the player applies force at the back of foot plate and the applied force (*F_A_*) exceeds the preset threshold (*F_T_*), the bat will move down. If the player applies force at the front of the plate and *F_A_* is less than −*F_T_*, the bat will move up. This is accompanied by up and down motions of the foot plate.

### 2.4. Pilot Study

Four healthy male adults (age: 24–28 years old; weight: 52–80 kg; height: 157–192 cm) and three male stroke patients who suffered from neurological foot drop for more than one year (age: 53–58 years old; weight: 63–83 kg; height: 152–176 cm) were recruited. The healthy participants did not have any known muscular or neurological disorder and were not under any influence of medication or alcohol. One of the stroke patients (Participant E) had grade 4 MRC (Medical Research Council) muscle scale, and the others (Participant F and G) had grade 1 MRC muscle scale. Patients under these two categories have the minimal ability to voluntarily move their feet. A participant with contracture of the ankle and foot, painful spasticity of foot muscles and/or involuntary muscle spasm or contraction due to nerve damaged was excluded from this trial. All participants were briefed on the experiment procedure and objective before giving their consent. This study was reviewed and approved by MUHREC (Monash University Human Research Ethics Clearance) (Ref. No. 10741) and the Institute of Research Management and Innovation of UiTM (Universiti Teknologi Mara) (Ref. No. REC/401/18).

Participants were instructed to sit on a tall chair, place their foot on the foot plate and relax their calf muscles. A monitor was placed in front of them to display the graphical user interface of the game (Figure 4b). A calibration was performed before the gaming session. In this calibration, the participants were requested to dorsiflex and plantarflex their foot voluntarily without any assistance. The RoM of the ankle and the amount of force generated to perform these motions were recorded and were set as the threshold. The RoM of the ankle was used to limit the RoM of the robot foot plate. The minimum force required to perform these motions was set as the force threshold (*F_T_*) and was used to register the patient’s intention. The duration of the game was 5 min. The actuator response and the final game score were measured and evaluated.

## 3. Results

**Figure 5:**
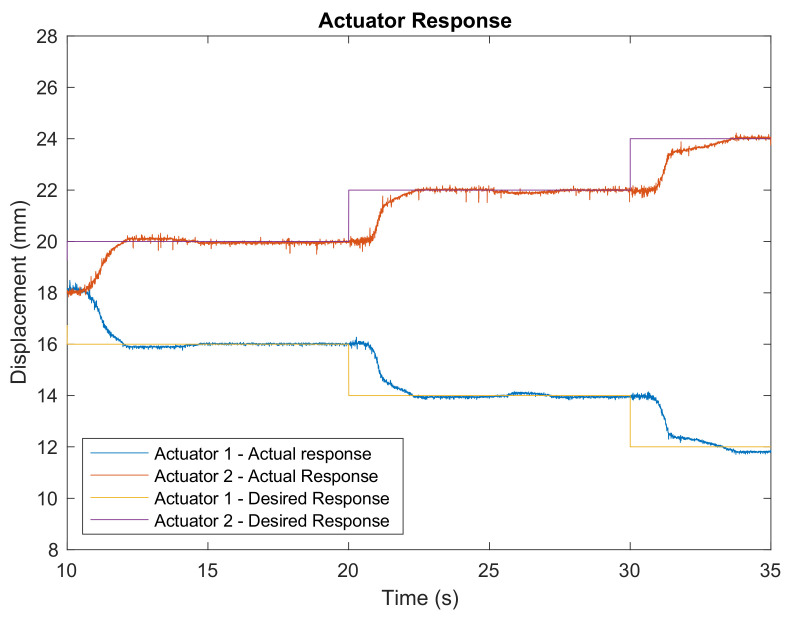
The actual and desired responses of Actuator 1 and Actuator 2.

The implementation of the two-state discrete antagonistic control on Actuator 1 and Actuator 2 is depicted in Figure 5. An incremental step input (2 mm per step) was supplied, and the actuator response was recorded. The average set point errors for Actuator 1 and Actuator 2 are 0.13 mm and 0.18 mm, respectively. These errors are acceptable as they only produce a deviation of approximately 0.24° in the foot plate orientation, which will not be felt by the participant. These results demonstrate the ability of the controller to manipulate the actuators antagonistically with minimal errors. The switching between the heating and cooling states occurred seamlessly. When one of the actuators was heated and contracted, the other was cooled and extended.

**Figure 6:**
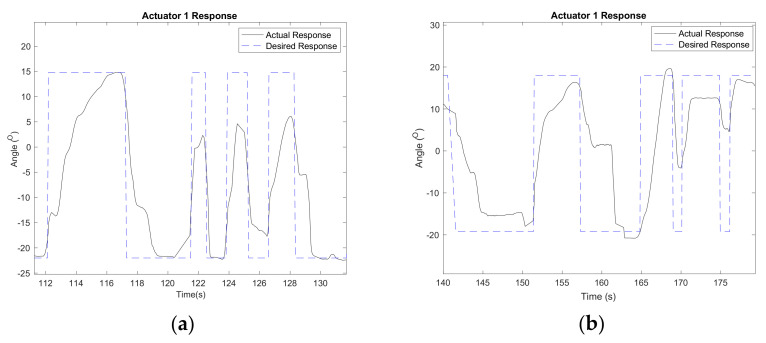
Actuator 1 response of the (**a**) healthy subject and (**b**) stroke patient during the game.

Figure 6 shows the desired and actual responses of Actuator 1 during the Pong game. The desired response was determined based on the participant’s intention to block the ball from entering the scoring region. It was derived from the measurement recorded by the load cell. When the participant dorsiflexed his foot and exerted force at the back of the foot plate, the robot extended Actuator 1 to the desired position of 14.46° and contracted Actuator 2. This motion tilted the foot plate upward and moved the bat up to hit the incoming ball. On the contrary, when the participant plantarflexed his foot, the robot tilted the foot plate downward and moved the bat down.

The actuators were able to generate displacement between 2.29 mm and 36.71 mm with minor deviations (Table 1). The largest average error was 0.37 mm, and the smallest average error was 0.02 mm, which corresponds to errors of 0.49° and 0.03°. These differences could be attributed to the incompatibility between the tall chair, the subject’s height and the robot, which reduced the effective displacement of the actuator and subsequently reduced the RoM of the robot foot plate.

The experiment validated the ability of the robot control to use foot motion as the main input to move the foot plate and the player’s bat in the game. The robot responded well to the intended foot motion during the game and changed the position of the foot plate when the foot applied sufficient force that exceeded the preset threshold. Both healthy participants and stroke patients did not manage to win the game but were able to complete it in 5 min.

**Figure 7:**
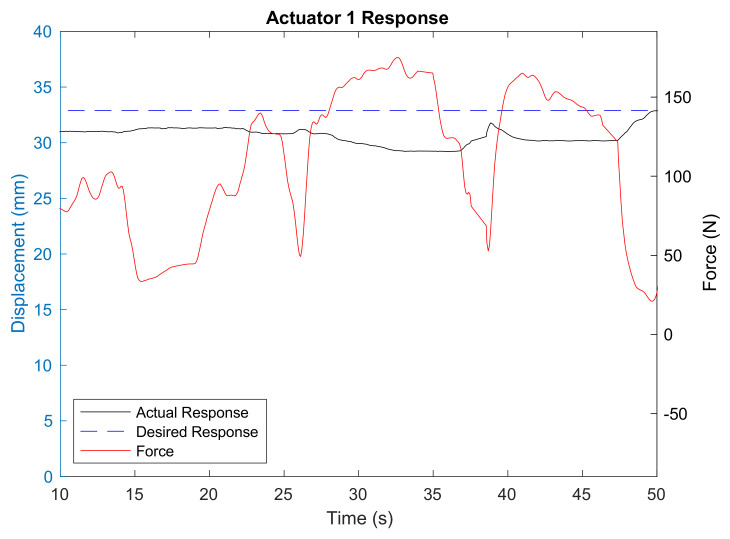
Actuator 1 response and force exerted by the patient.

The robot also displayed compliance to the external force exerted by the stroke patient (Figure 7). During the first 50 s of the treatment, the subject was trying to hold his foot in full plantarflexion position. Because of his condition, he could not fully control his foot and, therefore, failed to maintain it in the desired position. This caused the force to fluctuate between 0 N and 160 N. Although the robot resisted and slowed down Actuator 1 and Actuator 2 movements, it did not forcefully and rapidly rotate the foot plate as required, thus avoiding the risk of harming the patient’s ankle.

## 4. Discussion

The robot demonstrated its ability to provide active rehabilitation exercise using a nitinol wire actuator and the Pong game. Nitinol wire actuator offers several advantages. It is allergic-free and corrosive-free and has a high power-to-weight ratio [34]. It is also compliant with opposing forces and can adjust its position accordingly. This behavior creates tolerable interaction between patient and robot. It eliminates the risk of hyper-flexing the patient’s foot, thus making it a safe and compliant robot.

The proposed two-state discrete antagonistic control responded well to the user’s input during the game. The actuators extended and contracted as intended, and the switching between the two states, i.e., contraction and extension, occurred seamlessly. Changing from high voltage level to low voltage level during the contraction provides a means to fine-tune the actuator motion without overshooting the desired position. It also ensures that the wire does not overheat. Heating the nitinol wire beyond the allowable temperature can have negative consequences on the operation of the robot. It will take a longer time to lower the wire temperature during contraction, thus reducing the actuator response rate. Overheating the nitinol wire also shortens the overall lifespan of the wire. Nevertheless, the actuator control can be improved to provide variation in active rehabilitation exercises for different scenarios. One such control is the adaptive patient-cooperative control developed in [3], which was designed to enhance the effectiveness and safety of robot-assisted active exercise. This method also provides adaptive and robust trajectory tracking. Another type of control strategy that can be explored in the future is the impedance control proposed in [9,10]. This approach offers safe and effective interaction between humans and robots and is widely adopted in rehabilitation robots.

Nitinol is known for its slow response rate because it takes a longer time to lower its temperature and transforms from an austenite state to a martensite state. Several methods were proposed to increase the cooling rate of the nitinol wire [34]. Some used heat sink and heat conductive grease, while others immersed the wire in oil and water with glycol. However, they have their own limitations. Immersing the wire in oil or water poses a safety hazard to the user, especially when there is a leak in the system. Heat conductive gel can dissipate over time; therefore, it needs to be applied regularly. Forced convection offers a practical and economical way to improve the actuator response. It can be easily implemented by placing the fan at the right location, and it does not introduce additional safety hazards.

None of the players won the game, but in general, the healthy participants did better than the stroke patients. They were able to respond better to the incoming ball by flexing their foot voluntarily and changing the direction from plantarflexion to dorsiflexion and vice versa. On the contrary, stroke patients had difficulty controlling their feet naturally. This slowed down their response. However, as the patients’ recovery progresses, they will score better and closer to the average score of healthy participants.

Game-based training or intervention has been widely viewed as a means to promote adherence and cognitive capacity [35,36]. The use of various game elements such as points, difficulty levels, badges and storyline can enhance a patient’s intrinsic motivated behavior as it allows the patient to feel a sense of competence and accomplishment. Equipping the game with constant positive feedback can encourage the patient to understand what goes wrong in the previous round of the game and to allow them to improve their strategy or action in the next round. A study by [2] reported that a rehabilitation program that involves multiple active and engaging sessions could improve a patient’s condition. A review by [1] indicates similar results. The Pong game creates an interactive and engaging environment for the user. The difficulty level can be adjusted to suit the patient’s needs. The points/scores are recorded and can serve as an indicator of a patient’s recovery progress. Although the current study was limited to three stroke patients and one single treatment/session, it demonstrates the potential of the robot to be used in a multi-sessions program to continuously improve the patient’s ankle RoM, muscle force endurance and muscle strength.

The use of a single load cell is one of the unique features of this robot, considering that the other robots use several load cells, such as in [37]. The load cell measures the force exerted by the user when flexing their foot up and down, which is translated to the user’s intention to move the bat up and down in the game. This information is also correlated to the activation of the gastrocnemius and tibialis anterior muscles, which are responsible for the foot plantarflexion and dorsiflexion, respectively. This method eliminates the use of electromyogram (EMG), which is prone to crosstalk and requires a trained individual to place the electrode accurately to obtain reliable results. Nevertheless, this may not be sufficient if a more thorough investigation is required. Integrating an electromyogram (EMG) into the robot can provide a better overall outlook of the patient’s recovery progress. EMG sensors can be placed at the patient’s tibialis anterior and gastrocnemius muscles to monitor the activation of the muscle during the game. This can be coupled with the measurements collected by the load cell using the method proposed in [37] to determine if the muscles function appropriately to flex the foot up and down during the rehabilitation exercise.

The proposed robot has mechanical limitations. The current design only allows foot dorsiflexion and plantarflexion, while other robots can provide foot eversion and inversion, such as in [1]. The robot is also large and bulky; hence it will not be practical for mobile rehabilitation purposes.

## 5. Conclusions

Ankle rehabilitation robot offers several benefits. It can alleviate the physiotherapist’s workload. It can also provide a quantitative evaluation of a patient’s condition and recovery progress. This study proposes an ankle rehabilitation robot that uses a nitinol wire actuator and the Pong game to provide interactive and engaging exercise for post-stroke patients. The combination of these two elements is driven by the two-state discrete antagonistic control. It enables the manipulation of the nitinol wire actuator to tilt the foot plate up and down, and thus dorsiflexes and plantarflexes the foot during the game. The experimental results on the healthy participants and stroke patients were satisfactory. The robot was able to flex the foot based on the participant’s intention. When the user exerted a minimal downward force that exceeded the preset threshold, the robot plantarflexes the foot and vice versa. Although none of the participants won the game, it demonstrated the viability of this approach in rehabilitating the ankle.

## Figures and Tables

**Table 1:** Actuator response and final game score.

	Healthy Participants				Stroke Patients		
Subjects	A	B	C	D	E	F	G
Max. Ext. in mm	30.26	29.88	35.57	36.71	33.04	34.79	33.44
in degree	14.46	13.95	21.72	23.33	18.22	20.64	18.77
Max. Cont. in mm	2.29	3.32	5.42	5.57	4.25	12.81	13.72
in degree	−21.75	−20.38	−17.60	−17.41	−19.14	−8.07	−11.00
Max. Ext. Error in mm	0.24	0.18	0.13	0.31	0.05	0.37	0.25
in degree	0.32	0.24	0.17	0.41	0.07	0.49	0.33
Max. Cont. Error in mm	0.19	0.18	0.28	0.13	0.05	0.03	0.02
in degree	0.25	0.24	0.37	0.17	0.07	0.04	0.03
Computer Score	9	9	23	8	20	9	19
Player Score	1	4	2	3	5	1	3

Note: Max. Ext. and Max. Cont. are the maximum extension and contraction of Actuator 1, respectively.

## Data Availability

Not applicable.
